# How to Prepare Patients Receiving Antiresorptive Therapy for Tooth Extraction: A Narrative Review

**DOI:** 10.3390/ijms27177539

**Published:** 2026-08-23

**Authors:** Bartosz Bielecki-Kowalski, Lukasz Sokalski, Julia Majorczyk, Natalia Bielecka-Kowalska, Sebastian Klosek

**Affiliations:** 1 Department of Maxillofacial Surgery, Medical University of Lodz, 251 Pomorska St., 92-213 Lodz, Poland; bartosz.bielecki-kowalski@umed.lodz.pl; 2 Student Scientific Association, Department of Oral Mucosal and Periodontal Diseases, Medical University of Lodz, 251 Pomorska St., 92-213 Lodz, Poland; lukasz.sokalski@student.umed.lodz.pl (L.S.); julia.majorczyk@student.umed.lodz.pl (J.M.); 3 Student Scientific Association, Department of Maxillofacial Surgery, Medical University of Lodz, 251 Pomorska St., 92-213 Lodz, Poland; 4 Department of Oral Mucosal and Periodontal Diseases, Medical University of Lodz, 251 Pomorska St., 92-213 Lodz, Poland; sebastian.klosek@umed.lodz.pl; 5 Department of Oral Pathology, Medical University of Lodz, 251 Pomorska St., 92-213 Lodz, Poland

**Keywords:** MRONJ, BRONJ, prevention, tooth extraction

## Abstract

Bisphosphonates (BPs) and Denosumab (DMB) are antiresorptive agents (AA) commonly used in treatment of osteoporosis, multiple myeloma (8.0%), breast cancer (3.3%), prostate cancer (2.9%) and other malignancies (0.7%), bone metastases, and cancer-induced hypercalcemia. However, this therapy is associated with a significant risk of medication-related osteonecrosis of the jaw (MRONJ), particularly after tooth extraction. The aim of this narrative review was to summarize current evidence on measures aimed to reduce the risk of MRONJ after tooth extraction, and to critically discuss the strength of evidence supporting each of these measures. Articles published in the years 2013–2025 were reviewed using PubMed, Scopus, Web of Science databases. Available studies were categorized and compared with the use of selected prevention methods. The duration of the treatment, route of administration, dosage and type of AA have a significant impact on the risk of developing MRONJ. Antibiotic prophylaxis is considered in most published preventive protocols. However, the evidence supporting a single optimal regimen is limited. Patients from a high-risk group of MRONJ, demonstrating cancer, who were administered intravenous antiresorptive agents for longer than three years or zoledronic acid, or those with a history of jawbone necrosis or inflammation require prolonged antibiotic therapy (started before the procedure and continued up to 14 days after the procedure). Moreover, studies on the use of platelet-rich fibrin (PRF) and Concentrated Growth Factors (CGF) as a preventive measure have shown promising results in observational studies, along with antibiotic prophylaxis and optimal oral hygiene. Pentoxifylline with tocopherol and low-level laser therapy (LLLT) are recognized as potentially useful non-invasive preventions. However, evidence is limited due to the small sample sizes and heterogenous protocols. Vitamin D levels should be monitored, and oral supplementation should be considered if needed. AA therapy suspension prior to the surgical procedure must always be consulted with the attending physician. A multidisciplinary approach along with well-planned pre- and postoperative care is essential for safe tooth extraction in patients receiving AA therapy.

## 1. Introduction

Bisphosphonates (BPs) such as zoledronic acid (ZOL), Pamidronate and Ibandronate are a class of medications that affect bone resorption. This class is applied in treatment of diseases leading to bone loss such as Paget’s disease or osteoporosis. In addition, BPs may be recommended as an adjuvant treatment for cancer patients with multiple myeloma and bone metastasis mostly associated with breast, prostate (especially advanced breast cancer and bone metastasis) or lung solid tumors or for the treatment of cancer-induced conditions such as hypercalcemia [1,2,3]. Oral bisphosphonates are mainly used in treatment of osteoporosis and chronic metabolic diseases, whereas intravenous bisphosphonate are administered in conditions requiring a stronger antiresorptive effect, particularly in oncological patients and in bone pain associated with skeletal involvement [4].

Denosumab (DMB) is a human monoclonal antibody which blocks the RANK receptor located on osteoclast cells. It leads to suppression of bone resorption and may be used in treatment of osteoporosis or metastatic bone disease. There are other monoclonal antibodies available in treatment of osteoporosis such as a new one, Romosozumab (sclerostin antibodies). It also inhibits osteoclast cells, which results in decreased bone resorption but simultaneously, it increases bone formation by inhibiting sclerostin. However, there is not enough research confirming the role of sclerostin suppression in the development of MRONJ. A study conducted on mice by Nakashima et al. in 2024 showed that sclerostin deficiency is not related to osteonecrosis of jaw (ONJ) and Romosozumab is safe for a surgical approach. Yet, more studies are necessary in this area [5,6].

In fact, BPs and DMB have different mechanisms of action, but both cause the same effect. Research conducted in 2023 by Sayed SA et al. on 90 kidney transplant recipients demonstrated that subcutaneous administration of Denosumab and oral administration of Alendronate have a comparable improved effect on bone mineral density [7].

Cases of ONJ after BPs exposure were reported many times in publications and they are referred to as bisphosphonate-related osteonecrosis of jaw (BRONJ). Subsequently, an increasing number of cases of ONJ was reported in patients receiving other drugs than BPs, such as Denosumab or anti-angiogenic drugs. Therefore, the notion of medication-related osteonecrosis of jaw (MRONJ) is broader and refers to a various drug class [3].

MRONJ has been defined by the American Association of Oral and Maxillofacial Surgeons (AAOMS) and occurs as a consequence of the following elements: exposure of bone for more than 8 weeks, current or previous antiresorptive or anti-angiogenic drug therapy and absence of head and neck radiotherapy in medical history [6].

### 1.1. Risk Factors

BPs and DMB exposure may lead to MRONJ. Other risk factors may predispose individuals as well. Certain research was conducted to determine the most common risk factors associated with MRONJ. A meta-analysis performed by Srivastava et al. revealed the highest MRONJ rate in patients with multiple myeloma (8.0%), breast cancer (3.3%), prostate cancer (2.9%) and other malignancies (0.7%) [8]. The study conducted by Chang et al. showed that the most widespread diseases related to osteonecrosis due to antiresorptive drug administration are osteoporosis (48%), breast cancer (29%), multiple myeloma (10%), and prostate cancer (10%). What is more, the most common procedure after MRONJ diagnosis was tooth extraction (52%). Most of the patients were in their seventies (40%) and mainly females (82%). When it comes to BRONJ, it should be considered that most of osteoporosis patients were females, presumably because of post-menopausal osteoporosis [9,10]. Kizub et al. conducted a study on 6097 patients with breast cancer receiving adjuvant treatment with BPs. The patients were randomized to receive 4 mg of zoledronic acid IV monthly for 6 months, and subsequently every 3 months, 1600 mg of clodronate orally daily, or 50 mg of ibandronate orally daily for three years. It appeared that worse dental health (moderate/severe calculus, periodontal diseases) is associated with BRONJ, but dental plaque is not [11]. Apart from this, the mean duration of drug administration is also significant. There is a direct proportional relationship: the longer the intake of antiresorptive drugs, the higher the risk of developing MRONJ and BRONJ. Moreover, the mean duration is longer in patients with osteoporosis and shorter in patients with metastatic bone cancer [6,12]. It should be noted that in most quoted references there is a conclusion that not only the duration but also the dose and drug administration routes are significant. Patients receiving antiresorptive drugs intravenously are at much higher risk than those receiving the drugs orally. The condition of the oral cavity is a significant risk factor so chronic periodontal inflammation may compromise optimal oral health. Moreover, it affects the osteoclast numbers and function through different pathways. However, tooth extraction is the main local risk factor due to surgical trauma while processes occurring in bone are reduced by medications. Nevertheless, concomitant oral diseases, anatomical factors—if tooth extraction is supposed to be performed in the maxilla or mandible—age and sex, and other drugs such as corticosteroids are risk factors besides antiresorptive agent treatment and have to be noted in dental history [6]. All things should be considered before tooth extraction. Getting a specific dental history is required, including the exact onset and names of administered medications (corticosteroids, tyrosine kinase inhibitors (TKIs), monoclonal antibodies (bevacizumab), fusion proteins (aflibercept), mTOR inhibitors (everolimus), radiopharmaceuticals (radium 223), selective estrogen receptor modulators (raloxifene), and immunosuppressants (methotrexate)), systemic diseases (anemia, diabetes) and history of smoking tobacco. In some cases, it is not possible to perform tooth extraction, which is the safest option, prior to initiating a therapy. At that point, it is necessary to provide a safe dental procedure with proper prevention. It should be noted that the risk of developing BRONJ in osteoporosis patients following tooth extraction is still low, i.e., around 0.15%, and slightly higher in osteoporosis patients exposed to DMB, i.e., 1%. However, when it comes to cancer patients, the risk ranges from 1.6% to 14.8% [6]. Research performed by Kunihara et al. between 2016 and 2020, which consisted of investigating the incidence of osteonecrosis of jaw by antiresorptive agent dose, confirmed that cancer patients are at higher risk of ONJ than non-cancer patients. The incidence of ONJ in cancer patients treated with high dose of antiresorptive agents (AA) was 18 times higher than in osteoporosis patients treated with low dose of AA [13]. Depending on the patient’s condition, different medications are used. In treatment or prevention of osteoporotic fractures, Romosozumab can be recommended as it is associated with a significantly lower risk of MRONJ compared to BF such as Alendronic acid, Pamidronic acid, Ibandronic acid, Risedronic acid or Denosumab (RANKL inhibitor). Zolenronic acid and Denosumab demonstrate the highest risk of MRONJ [10]. Consequently, these two groups should be considered separately while it comes to prevention protocol before tooth extraction. To determine the risk, the main factors should be outlined: treatment regimen (low or high dose), treatment duration (longer or shorter than 3 years) and any MRONJ risk factors (smoking, corticosteroids, poor oral hygiene, etc.) [14] (Table 1).

Articles published in the years 2013–2025 were reviewed using PubMed, Scopus, Web of Science databases. The following keywords and their combinations were used: “MRONJ”, “BRONJ”, “medication-related osteonecrosis of the jaw, “bisphosphonates”, “denosumab”, “tooth extraction”, “prevention”, “antibiotic prophylaxis, “platelet-rich fibrin”, photobiomodulation”, “pentoxifylline”, vitamin D”, “drug holiday”. Available studies were categorized and compared based on selected prevention methods.

Inclusion criteria: Original clinical studies, systematic reviews, meta-analyses, and internationally recognized clinical guidelines regarding prevention of MRONJ before or after tooth extraction in adult patients receiving antiresorptive therapy.

Exclusion criteria: Case reports including fewer than five patients, conference abstracts, articles focused exclusively on management of established MRONJ without a preventive perspective, and studies concerning osteoradionecrosis (ORN) unrelated to antiresorptive therapy (Table 1).

### 1.2. Beta-C-Terminal Telopeptide

Beta-C-terminal telopeptide (CTX), also known as C-terminal telopeptide of type I collagen, is a specific biomarker released during bone resorption. CTX due to osteoclasts activity during collagen I degradation is released into circulation via protease cathepsin K activity. CTX value, serving as an additional indicator, used to be considered helpful in predicting probability of MRONJ in patients who are receiving antiresorptive medication and need an oral surgery procedure. Risk level associated with CTX value: high risk—below 100 μg/mL, moderate risk—between 100 and 150 μg/mL, and low risk—above 150 μg/mL. Patients who developed MRONJ demonstrated CTX below 100 μg/mL. Meta-analysis showed that CTX seems to be an insufficient MRONJ risk marker. Additionally, the 150 μg/mL cut-off does not correlate with increased prevalence of osteonecrosis [15,16].

## 2. Discussion

Preventive measures can be grouped into two categories: core measures, supported by consistent evidence and international guideline consensus and applicable to essentially all patients undergoing tooth extraction during antiresorptive therapy, and adjunctive measures, whose efficacy remains uncertain or is supported only by heterogeneous or low-level evidence, and which should be considered on a case-by-case basis rather than applied universally.

### 2.1. Fundamental Measures

The following measures are consistent with international position papers and practice statements (AAOMS 2022, Nicolatou-Galitis et al.) and should be considered standard care in all patients [6,14].

#### 2.1.1. Risk Stratification

Risk stratification prior to any invasive procedure is based on the underlying indication for antiresorptive therapy (oncological or osteoporotic), route of administration (intravenous or oral), specific agent (e.g., zoledronic acid and denosumab), cumulative dose, duration of treatment, concomitant systemic therapy, comorbidities and any previous history of MRONJ [6,9,11,12].

#### 2.1.2. Dental Rehabilitation Prior to Initiating Antiresorptive Therapy

Dental rehabilitation before antiresorptive therapy includes extraction of non-restorable teeth, treatment of active periodontal or periapical disease, and adaption to misaligned prostheses [6,14,17].

#### 2.1.3. Local Infection Control and Antiseptic Irrigation

According to AAOMS, maintaining good oral hygiene reduces the incidence of MRONJ. Moreover, patients should optimize dental health as early as before initiation of antiresorptive therapy, especially cancer therapy. All teeth that could cause a problem should be treated or extracted before antiresorptive therapy. If any dental treatment is undertaken during antiresorptive therapy, antimicrobial preoperative and postoperative mouth rinses should be used in both oncological and non-oncological patients [6]. Patients are advised to use 0.2% chlorhexidine gluconate mouth rinses for 7 days following extraction to prevent risk of development of MRONJ/BRONJ [18]. Oral infections significantly contribute to bone necrosis. Patients under bisphosphonate therapy with a higher decayed, missing, and filled teeth index were correlated with higher osteonecrosis staging [17]. Additionally, regular dental check-ups are indicated for patients who had recommended preventive dental care.

#### 2.1.4. Atraumatic Extraction Technique, Alveoplasty, and Primary Wound Closure

The surgical procedure itself should be as atraumatic as possible, minimizing the trauma to the alveolar bone and surrounding soft tissues. After tooth removal, sharp bone margins should be smoothed (alveoplasty) to reduce the risk of mucosal perforation and secondary contamination of the socket. Primary wound closure should be achieved whenever possible to protect the underlying bone from oral microflora and mechanical irritation [14,19,20,21,22].

#### 2.1.5. Careful, Structured Postoperative Follow-Up

Structured postoperative follow-up is essential in order to detect delayed healing at an early stage. It is typically planned for 1, 2, 4 and 8 weeks after extraction [6,14].

### 2.2. Adjunctive Measures

#### 2.2.1. Antibiotic

According to the Polish Dental Association and the National Program of Antibiotic Protection, the first dose should be taken one day before the procedure, and the administration should continue for the subsequent three days. If risk factors are present, such as intravenous bisphosphonate administration, treatment duration exceeding three years, application of zoledronic acid, or a history of inflammation or necrosis of the jawbone, antibiotic therapy may be extended up to 14 days. However, the optimal duration remains uncertain and should be individually tailored to the patient depending on their risk profile. For most patients, amoxicillin with clavulanic acid is prescribed, at a dose of 1000 mg (875 mg of amoxicillin and 125 mg of clavulanic acid) taken every 12 h. For those allergic to penicillin, clindamycin is recommended as an alternative, at a dose of 300 mg every 8 h [23]. According to an Italian scoping review, antibiotic prophylaxis is recommended in patients who will be subjected to extraction and are at risk of MRONJ. Amoxicillin was most commonly used, either alone or with clavulanic acid, with metronidazole added only in eight out of 36 analyzed studies. The median antibiotic duration was 1.5 days pre-extraction, 5.5 days post-extraction, and 7 days in total [24].

In one particular study involving 760 patients under antibiotic prophylaxis, beta-lactam antibiotics were primarily used (Amoxicillin 875 mg + 125 mg orally or Sultamicillin tosilat 1.5 g, i.v.), whereas patients with penicillin hypersensitivity were administered clindamycin (Clindamycin 600 mg). Three groups of patients were examined: Group 1 (719 extractions) received intravenous antibiotics for 7 days, Group 2 (126 extractions) received oral antibiotics for 14 days, and Group 3 (298 extractions) received oral antibiotics for 7 days. Sixty percent of patients were diagnosed with cancer, while the remaining were treated for osteoporosis. Regarding AA therapy, 72% of patients received bisphosphonates, 20% denosumab, and the remaining number—both. Beta-lactam antibiotics were used in 85% of patients, and the success rate was 93.4%. Clindamycin was administered in 12% of cases, with a 92.4% success rate, and other antibiotics (3%) contributed to a 95% success rate. The therapy success rate (complete mucosal recovery after 3 months) was 94.3% in Group 2, compared to 93% in Group 1 and 92.9% in Group 3. Yet, Group 1 included a higher proportion of high-risk patients (64%) (AA therapy according to the protocols for the therapy of malignant disease with bone metastasis or multiple myeloma); the percentage of high-risk patients in Groups 2 and 3 was 47%. Low-risk patients (e.g., treated for osteoporosis) showed a markedly better success rate, compared to the high-risk group of patients—98% and 90%, respectively. These results suggest a potential benefit of extending antibiotic prophylaxis by 14 days. Differentiating between infected extraction sites and MRONJ is crucial for accurate differential diagnosis [25].

According to Cabras et al., the proof to justify the routine use of antibiotic prophylaxis is insufficient. Only one retrospective study reported a higher risk of MRONJ in patients who were not exposed to antibiotics [26].

Prolonged antibiotic prophylaxis, particularly regimes extended up to 14 days, should be carefully considered due to the risk of adverse effects and antimicrobial resistance. Broad-spectrum agents such as amoxicillin–clavulanate and metronidazole are associated with gastrointestinal side effects and disruption of oral and gut microbiota. From a public health perspective, unnecessarily long courses contribute to the global burden of antibiotic resistance. Therefore, the decision on antibiotic duration should be individualized: short-course regimens (1–7 days) appear reasonable in low-risk patients undergoing simple extractions, whereas extended prophylaxis should be considered in high-risk patients [20,21,22].

#### 2.2.2. Autologous Platelet Concentrates

Autologous platelet concentrates (APCs), thanks to their immunomodulatory properties, providing platelet factors and enhancing of the angiogenesis promotion can be helpful in prevention and treatment of MRONJ. In a systematic review across 43 studies, MRONJ was rare, observed only in 12 cases (1%) of 1219 extractions, including 786 with APCs. MRONJ developed in 0.9% of the sites where autologous platelet concentrates were applied (7/786) and in 1.2% of the sites, MRONJ was managed without APCs (5/433), indicating only a minimal and statistically non-significant absolute risk reduction associated with APC use (*p* = 0.7634) [27]. APCs are mostly represented by platelet-rich fibrin (PRF) and PRP (platelet-rich plasma). PRP and PRF can improve healing processes by reducing inflammation and accelerating wound recovery, thus potentially minimizing the MRONJ risk. PRP and PRF block the entrance for bacteria by closing a post-extraction cavity. In addition, they support healing processes by delivering growth factors and cellular components. Also, bone regeneration can be performed well by an efficient healing process and quicker closure of the wound [28]. PRF is a promising adjunctive factor in managing bisphosphonate-induced complications. The successful treatment rate in the study conducted by Parise et al. showed that the success ratio for oncological patients who underwent L-PRF (Leukocyte- and platelet-rich fibrin) prophylaxis compared to the non-receiving L-PRF control group was 100% to 57%. Both groups were administered intravenous zoledronic acid for oncological treatment. All patients received 500 mg of amoxicillin and 400 mg of metronidazole. The therapy started 7 days before the surgery and continued every 8 h for 7 days after the extraction. In addition, rising with 0.12% alcohol-free chlorhexidine gluconate twice a day for 1 min was recommended [29]. In the observational study by Besi et al., no cases of MRONJ were reported among patients treated with L-PRF, while MRONJ occurred in the high-risk group managed without L-PRF. Notably, the study protocol did not include systemic antibiotic prophylaxis; the patients were instead instructed to use a 0.2% chlorhexidine mouthwash [19]. In a particular study, MRONJ developed in the control group, in which tooth extractions were performed without PRF insertion, while no cases of MRONJ were reported in the study group treated with PRF after extraction. The patients who developed MRONJ included individuals with oncological disease and osteoporosis and they were treated with bisphosphonates, including ibandronate and alendronate. In the PRF-treated group, patients also presented systemic risk factors, including osteoporosis and oncological disease. Most of them were treated with alendronate, while one patient received denosumab. However, the specific type of PRF used in this study was not specified. The perioperative protocol included systemic antibiotic prophylaxis with amoxicillin/clavulanic acid administered at a dose of 1 g every 12 h for 5 days before and 5 days after the dental procedure, as well as metronidazole at a dose of 250 mg every 12 h for 2 days before and 2 days after the procedure [30]. In a study performed by Alrmali et al., all patients demonstrated successful healing after PRF insertion. The patients have used zoledronic acid for more than 1 year, more than 3 years and 7 years; four of them were treated for breast cancer metastasis [31]. A systematic review on the total number of 697 extractions in patients who received intravenous BPs therapy (mostly zoledronic acid) and preventive PRP showed that only seven patients (0.7%) of the above total number demonstrated ONJ. Eight articles reported prevention with APC: seven with PRP, and one with L-PRF and PRF [32]. Tolentino et al. performed a systematic review of management of MRONJ and reported complete healing in 65 patients (80.2%) and significant symptom improvement in 14 (17.3%) using PRP [33]. In the systematic review by Nowak et al., APCs were associated with an 86.13% effectiveness rate in MRONJ prevention [34,35]. Observational data suggest that APCs may support post-extraction healing, but pooled data do not consistently demonstrate a statistically significant reduction in MRONJ incidence [23].

Nowadays, new solutions are being studied. They include composite hydrogel scaffold consisting of methacrylated gelatin (GelMA), methacrylated heparin (HepMA) and PRF. Hep/GelMA-PRF solution can solidify into hydrogel in the presence of ultraviolet light. It has excellent physical properties and ensures the suitabile microenvironment for new bone formation, which effectively prevents the invasion of foreign pathogen. So far, research has been performed in rats receiving zoledronic acid; however, this approach may also prove beneficial for human patients in the future. [36].

Another recent development is the concept of antibiotic-loaded PRF (AL-PRF) in which antibiotics (e.g., amoxicillin, clindamycin, metronidazole, or gentamycin, linezolid, vancomycin) are incorporated into the fibrin matrix at the time of preparation. AL-PRF combines the biological benefits of PRF (growth factor release and support of local tissue healing) with sustained local antimicrobial activity, demonstrated in vitro, potentially reducing the need for systemic antibiotics in selected patients and offering targeted antibacterial effect against oral pathogens at the surgical site. Studies on the use of AL-PRF in MRONJ prevention are still in their preliminary, in vitro stage. AL-PRF is a promising path for future research, particularly in patients at risk, in whom minimizing of systematic antibiotic exposure is desirable [37,38].

#### 2.2.3. Photobiomodulation and Laserotherapy

Low-level laser therapy (LLLT), also known as photobiomodulation (PBM), stimulates osteogenesis and decreases the healing time of bone tissue [39]. Red and infrared wavelength photons react with photoreceptors in cells and increase mitochondrial ATP production [40]. Additionally, it improves collagen deposition and reduces pain [41]. Application of 1.2 J/cm^2^ and 808 nm wavelength laser therapy can prolong the survivability of alendronate-treated osteoblasts and increase RANKL and M-CSF expressions in these cells; however, osteoprotegerin (OPG) is not significantly affected [42]. Significantly higher expression of osteocalcin occurred in a group of rats which received Nd:YAG laser prevention BRONJ (ZOL and dexamethasone) after tooth extraction [43]. A systematic review by Tolentino et al. showed complete healing in 98 of 246 patients (39.8%) and symptom improvement in 158 (64.2%) after low-intensity laser therapy in MRONJ management [33]. According to a meta-analysis and randomized studies conducted over the last two decades, LLLT can be used in patients treated by chemotherapy or radiotherapy for advanced head and neck cancer, which suggests that LLLT is safe for oncological patients [44]. Systematic reviews reveal that evidence is not strong enough to define a particular protocol. Nevertheless, it has been become more popular in the last few years [39,45].

Vescovi et al. carried out two studies on a total of 589 extractions using photobiomodulation. In the first study, in only five cases out of 217 patients, minimal bone exposure was observed, while the remaining sites healed properly. LLLT was administered using Nd:YAG (1064 nm wavelength Fidelis Plus, Fotona^®^, Ljubljana, Slovenia; power: 1.25 W; frequency: 15 Hz; fiber diameter: 320 μm; power density: 7 J/cm^2^), with five applications of 1 min each, once a week for 6 weeks. Additionally, antibiotic was administered (amoxicillin, 2 g per day, started 3 days before tooth extraction and continued postoperatively for 14 days) and mouthwash with chlorhexidine and hydrogen peroxide was used three times per day. In these five cases with less than 3 mm bone exposure, management was based on combined antibiotics therapy (amoxicillin, 2 g per day, and metronidazole, 500 mg per day) and LLLT. Residual bone fragments were curetted and vaporized using Er:YAG laser (2940 nm, 250 mJ, 20 Hz, VSP, Fluence 50 J/cm^2^, Fidelis Plus, Fotona^®^, Ljubljana, Slovenia). Wound closure was obtained within 2 months [20]. In another study by Vescovi et al., only two cases of MRONJ occurred among 36 patients undergoing 82 extractions. This time, the patients had previously been affected by MRONJ. Nd:YAG photobiomodulation, applied in five sessions of 1 min each, and antibiotic was administered from day 3 before extraction to 2 weeks after the operation [21]. Şahin et al. achieved complete mucosa healing in all patients using the preventive protocol with Nd:YAG laser (the same devices as Vescovi et al. used, 1–2 mm from the tissue for 1 min) on days 2, 5, 7, 10, 14, 21 and L-PRF (2700r rpm for 12 min in 10 mL tubes) was placed into the extraction socket [22]. Vescovi used 2 g amoxicillin and Şahin prescribed 1 g amoxicillin with clavulanic acid.

Tartatori et al. revealed that no signs of MRONJ were observed after at least 6 months of follow-up, in 18 patients who were administered photomodulation (660 nm, 321 J/cm^2^, 27 J in total) after extractions [46].

In a preclinical mouse model study, Zheng et al. administered ZOL and low-level laser therapy (Velure S9 980 Laser, 980 nm wavelength, on days 0, 2 and 4 following the operation). Follow-up showed complete wound healing (mucosal covering), ample new bone formation, greater osteoclasts formation and more collagen deposition in the extraction sockets. A similar effect was observed in the control group without LLLT and ZOL use, with only extraction. Meantime mouses which have taken ZOL with no LLLT prevention demonstrated incomplete mucosal healing, lack of epithelial coverage, exposed bone, necrotic bone with empty lacunae and fewer osteoclast formations [47]. Özalp et al. performed a preclinical mouse model study using LLLT (gallium–aluminum–arsenide, 808 nm wavelength, diode laser, Epic 10, Biolase) combined with ozone therapy on days 3, 5, 7 and 10 after the operation. The LLLT + ozone group showed significantly greater bone formation than the control group, whereas the LLLT-only and ozone-only groups did not demonstrate statistically significant differences [48].

There is a wide variation regarding parameters, duration of the therapy and the dose between studies. Therefore, choosing one specific protocol is difficult. The wavelength in LLLT ranges between 600 and 1100 nm. Reis et al. pointed at the Arsenide–Gallium–Aluminum laser, with 830 nm wavelength, considering it the most common laser in photomodulation which improves wound regeneration. Additionally, fibrin sealant was the most commonly used form of fibrin, followed by PRF and L-PRF [49]. Hendler et al. compared the 830 nm and 660 nm wavelengths and revealed that the former wavelength is more effective than the latter wavelength [50]. However, Nd:YAG laser therapy appeared to be excellent as a prevention tool after tooth extractions. However, it may generate more heat so doses should be carefully adjusted (five times per 1 min) [20,21].

#### 2.2.4. Pentoxifylline and Tocopherol

Pentoxifylline and tocopherol have been found useful for in MRONJ management. Pentoxifylline is a medicine which inhibits erythrocyte phosphodiesterase. It lowers blood viscosity, improves microcirculation and tissue perfusion [51]. Tocopherol (alpha-tocopherol/vitamin E) antioxidant was previously applied in osteoradionecrosis treatment. It modulates the expression of genes and partially inhibits transformation of the growth factor-beta1 [52]. The PENTO protocol, which involves prescribing these medications, is reported to be well tolerated, causing only minimal side effects. In addition, in comparison with other non-surgical treatments it is cost-effective [53,54]. The drug is mostly prescribed at a dose of 400 mg pentoxifylline and administered two times a day. Dose adjustment for tocopherol varies, with common regimens being 500 mg twice a day, 400 mg twice a day, or 400 IU twice a day. According to the abovementioned studies, pentoxifylline and tocopherol are beneficial in the non-invasive management of MRONJ, contributing to a significant reduction in lesion size in all patients. Bone exposure was completely resolved in 60.86% of patients (14 out of 23), with partial resolution in 30.43% of patients. Prophylaxis with pentoxifylline and tocopherol reduces the severity of injuries, is well tolerated, and has high patient compliance. MRONJ occurred in only 9.4% of extracted teeth.

Using pentoxifylline in combination with tocopherol seems to be clinically reasonable. However, there is a lack of randomized studies, large sample sizes and lack of homogeneity between the articles [55].

#### 2.2.5. Vitamin D

Vitamin D deficiency is a widespread problem not only in prevention of MRONJ and BRONJ but also in general health aspects and for the whole population. Around 30% of young people and 80% of older people have vitamin D insufficiency <20 ng/mL. It is imperative that levels >32 ng/mL be a prophylactic method of osteoporosis and cancer. All people should supplement vitamin D in appropriate doses depending on the degree of deficiency and it should especially be controlled in patients receiving antiresorptive drug therapy [56]. Some studies have illustrated the association between a low level of vitamin D and oral pathology. Vitamin D deficiency relates to a higher risk of severe chronic periodontitis in adults as well as a higher risk of caries in children. In addition, it is related to occurrence of osteomalacia in jaws which might be crucial for MRONJ pathogenesis. Thus, it can be concluded that receiving antiresorptive drugs is probably not the main cause of MRONJ, but osteomalacia probably is. So that vitamin D supplementation has a key influence in prevention of ONJ. Safe levels of vitamin D should be investigated more thoroughly. It is advisable to bear in mind that hypovitaminosis D is commonly present in cancer patients, which more often develops MRONJ [57].

Additionally, a study conducted by Demircan et al. on patients with BRONJ showed that they exhibited a statistically higher level of Parathormone and a lower level of vitamin D [58]. As a conclusion, vitamin D supplementation on patients under Bps treatment should be considered as it might prevent deficiency and decrease the risk of fracture.

When it comes to Denosumab, vitamin D supplementation can also be prescribed. A study carried out by Suzuki et al. demonstrated that in patients receiving Denosumab and Eldecalcitol (active vitamin D analog) tablets of 0.75 μg daily, mineral bone density was improved more effectively than in patients administered Denosumab and 200 IU of Cholecalciferol [59].

Lorenzo-Pouso et al. reveal a hypothetical pathway in which vitamin D supplementation may prevent MRONJ. The supplementation prevents hypocalcemia and hyperparathyroidism which are responsible for bone turnover suppression, angiogenesis inhibition and impaired immune responses. What is more, it was mentioned that one of local triggers of MRONJ is inflammation. Vitamin D intake reduces pro-inflammatory cytokines (IL1, IL2, TNFalfa). Vitamin D reduces Porphyromonas Gingivalis adhesion and infectivity in human gingival epithelium and periodontal ligament cells, which may help in treatment of periodontal diseases [60,61]. This may improve periodontal tissue stability and reduce chronic oral inflammation, and the latter is considered one of the local triggers of MRONJ. Moreover, adequate vitamin D levels may support immune response, angiogenesis and physiological bone remodeling, which may promote proper post-extraction healing. Consequently, vitamin D supplementation may indirectly reduce susceptibility to jawbone necrosis in patients receiving antiresorptive therapy.

#### 2.2.6. Drug Holiday

Many studies were conducted to determine whether drug holiday is a proper prevention of MRONJ or not. However, it is still inconclusive. First of all, both the dentist and attending physician should be aware of medical events of taking or not taking antiresorptive agents (AA) and make that decision in consultation with each other. There are differences between Denosumab and BP mechanisms.

BPs are permanently and strongly connected to hydroxyapatite found in bones and can stay there even for years. In contrast, denosumab circles in blood bonding selectively to RANKL receptors and is not found in a bone. This leads to the conclusion that drug holiday on Denosumab treatment may be more convenient because of the time of presence in the body. When it comes to DMB, according to the AAOMS statement tooth extraction can be performed 3–4 months after discontinuation, when the inhibition of osteoclast decreases. However, some research shows it takes 12 months to stop suppression of osteoclasts in patients receiving high doses of denosumab [62].

In the randomized control study conducted by Ottesen et al. all patients were given high doses of AA, including both Denosumab and BPs. They have had tooth extraction performed 4 months after the last dose of AA but drug suspension was not able to prevent them from developing MRONJ. Approximately 30% of patients developed MRONJ; all of them had been under Denosumab treatment before. Results suggest that drug holiday from a high dose of AA does not prevent from developing MRONJ. What is more, the group which had drug holiday that presented with aggravated medical condition can be compared with the group which continued the treatment [63].

Park et al. conducted a clinical observation study evaluating the association between discontinuation of intravenous bisphosphonates and the risk of BRONJ after tooth extraction. The research showed that the longer the period since the last dose of iv bisphosphonates is, the lower the risk of developing BRONJ, particularly if the interval exceeded 90 days. Results suggest that temporary discontinuation of intravenous bisphosphonate therapy before dental surgery may lower the risk of BRONJ. However, extended discontinuation of medication therapy may increase the risk of osteoporotic fractures [64].

One of the most recent research projects carried out by Otto et al. on animals proves that a drug holiday combined with antibiotic prophylaxis and smoothening of sharp edges prevent MRONJ. The study involved four groups: negative control (1), group of minipigs under ZOL therapy without drug holiday and wound management (2), a group of minipigs under ZOL therapy without drug holiday but wound management and antibiotics were included (3), and a group of minipigs under ZOL therapy while tooth extraction was performed 6 weeks after the last dose of ZOL with proper prevention (antibiotics + wound management) (4). Approximately 83% of cases in Group 3 developed MRONJ in the area of tooth extraction while in Group 4, the rate was considerably lower, i.e., 40%. So, despite BPs long half-life in bone, drug suspension may be a clinically relevant element in the MRONJ prevention protocol [65]. Another study was conducted on rats by Zandi et al. to assess the effectiveness of drug holiday of Zoledronate. It showed that the incidence of ONJ decreased when drug holiday was growing. In other words, over 4 months of drug holiday, the incidence of osteonecrosis was 45% and over 1 month of drug holiday, the value was 75%. What is more, the longer the drug holiday was, the higher the percentage of new bone formation; over 4 months of drug holiday, the value was 50% [66]. On the other hand, drug holiday issue still needs to be analyzed more thoroughly, and specialist should consider medical events individually.

Furthermore, drug holiday introduced in patients with osteoporosis receiving bisphosphonates can lead to fractures that may be more dangerous than performing the tooth extraction during treatment. There is a different effect of drug holiday when it comes to the type of bisphosphonates. Post-fracture mortality is higher in people who discontinue Risedronate than Alendronate administered orally [67].

To conclude, if the oral surgeon and attending physician have decided to suspend AA therapy to perform a tooth extraction, it should be discontinued 3–4 months before the procedure for DMB and 4 months before the procedure for intravenous BPs, such as Zoledronate acid. Treatment in all cases should be resumed within 6–8 weeks following the surgery to ensure proper bone healing.

Evidence on drug holiday is contradictory. There is no consensus among international guidelines (AAOMS, ASBMR, EAO). The decision must always be individualized and made in consultation with the attending physician, balancing the risk of MRONJ against the risk of disease progression and osteoporotic fractures. Drug holiday should not be presented as a universally protective measure [63,64,65].

#### 2.2.7. Hyperbaric Oxygen Therapy

Hyperbaric oxygen therapy (HBO) is often used successfully as an adjuvant treatment of MRONJ/BRONJ [68,69,70]. The therapy is advantageous over antibiotics in prevention of osteoradionecrosis and its implementation is also considered before and after tooth extraction in such patients [71]. One study indicated that the therapy relieved pain and decreased the size and number of lesions in 75.6% of 41 patients receiving HBO [33]. In a study conducted by Sao-Shen Liu et al., rats exposed to zoledronic acid underwent immediate hyperbaric oxygen therapy after tooth extraction as a prophylaxis of osteonecrosis. The method was able to reduce the development of MRONJ in rats [72]. What is more, a study performed by Liao et al. on dogs in 2020 confirms that HBO is able to reduce bone resorption by osteoclast inhibition and promote early bone formation [73]. Nevertheless, there are too few reports on humans to determine the effectiveness of such a method for medication-induced osteonecrosis. To conclude, this topic still requires further examination. However, HBO before or immediately after tooth extraction may become a part of MRONJ/BRONJ prevention protocol in the future. According to ASCO guidelines for prevention of osteoradionecrosis in patients with head and neck cancer treated with radiation therapy, routine-use HBO in case tooth extraction is not recommended due to limited evidence supporting the introduction of the above method. However, prophylactic HBO does improve percentage of dental socket healing and may help a small subset of patients at elevated risk of ORN, especially those patients in whose substantial volume of mandible/maxilla was >50 Gy [74].

To systematize the collected information, Table 2 systematizes the collected information by presenting the strength of evidence for each preventive measure, whereas Table 3 describes prevention methods according to risk (Table 2 and Table 3).

## 3. Conclusions

Medication-related osteonecrosis of the jaw remains a clinically important complication in patients receiving antiresorptive or anti-angiogenic therapy, particularly when invasive oral procedures are required. Current evidence indicates that the risk of MRONJ is not determined by a single factor, but results from the interaction between the type of medication, indication for treatment, duration and route of administration, systemic comorbidities, local inflammatory status, and the extent of surgical trauma. Therefore, prevention should not be based on one universal intervention, but preferably on individualized risk stratification and a combined pre-, intra-, and postoperative protocol.

Patients treated for malignant diseases, especially those receiving high-dose intravenous bisphosphonates or denosumab, represent the highest-risk group. In contrast, patients treated orally for osteoporosis generally present a lower absolute risk; however, this risk increases with treatment duration, poor oral health, concomitant systemic diseases, corticosteroid use, and previous inflammatory or necrotic jawbone lesions. These differences justify categorization of patients into low- and high-risk groups before tooth extraction. Such stratification allows clinicians to avoid overtreatment in low-risk patients while maintaining a more cautious approach in oncological or otherwise medically compromised individuals.

Oral hygiene and local infection control appear to be fundamental elements of MRONJ prevention. For this reason, dental optimization before the initiation of antiresorptive therapy remains the safest approach. Antibiotic prophylaxis was included in most of the analyzed preventive protocols, although the evidence does not allow a single regimen to be identified as superior. Longer postoperative prophylaxis may be beneficial in selected high-risk patients, but routine prolonged antibiotic use should be balanced against the risk of adverse effects, antimicrobial resistance, and individual patient factors.

Among adjunctive biological methods, APCs, particularly PRF and L-PRF, may provide additional benefit by supporting socket closure, stabilizing the clot, delivering growth factors, and improving early soft tissue healing. However, systematic data remain heterogeneous and the absolute risk reduction is not always statistically significant. Therefore, APCs should be interpreted as a promising adjunct rather than an isolated preventive method. PBM shows a similar profile—clinical studies have reported favorable healing outcomes, but because laser therapy was rarely evaluated as a standalone intervention and protocols remain highly heterogenous, its independent preventive effect cannot yet be defined. PBM may nevertheless be considered as an adjunctive method, especially in patients with delayed healing risk, provided that oncological safety and local tumor status are carefully considered. Vitamin D further plays an indirect role in MRONJ pathogenesis. Assessment and correction of deficiency appear clinically justified as a part of MRONJ risk management. However, current evidence is insufficient to confirm that vitamin D supplementation alone prevents MRONJ.

The role of temporary discontinuation of antiresorptive therapy remains controversial. Particularly, oncological patients receiving high-dose therapy did not demonstrate a clear preventive effect and raised concerns about worsening systemic disease control. Any decision to interrupt antiresorptive therapy must therefore be individualized and made in consultation with the treating physician, with careful consideration of both dental risk and the risk of skeletal-related events or disease progression. Pentoxifylline with tocopherol represents another potentially useful adjunctive approach, but lack of randomized studies, small sample sizes, and heterogeneity of protocols limit the strength of any recommendation. Its preventive use should therefore be considered experimental or reserved for selected high-risk cases. Hyperbaric oxygen therapy has been investigated mainly as an adjunctive treatment for osteoradionecrosis and established MRONJ. Moreover, current recommendations for osteoradionecrosis prevention do not support routine hyperbaric oxygen therapy in all patients undergoing dental extraction, although it may be considered in selected high-risk cases and remains a potential area for further research.

In the authors’ view, based on currently available evidence, a rational approach to MRONJ prevention appears to be a multimodal, individualized strategy based on patient risk assessment rather than a single standardized protocol. In low-risk patients, careful extraction technique, infection control, antiseptic rinses, and short antibiotic prophylaxis may be sufficient. In high-risk patients, especially those treated for malignant disease or receiving high-dose intravenous antiresorptive therapy, a more comprehensive approach should be considered. This may include prolonged antibiotic coverage, atraumatic extraction, smoothing of sharp bone edges, primary closure whenever possible, use of PRF or L-PRF when appropriate, adjunctive PBM, strict postoperative monitoring, and interdisciplinary consultation regarding possible modification of systemic therapy. Throughout this review, recommendations directly supported by international guidelines (AAOMS; Nicolatou-Galitis et al.) [6,14] are clearly identified, whereas suggestions based on lower-level evidence are presented as the authors’ interpretation of the current literature rather than formal recommendations.

No single preventive method provides complete protection against MRONJ. The current literature supports the use of combined strategies rather than isolated interventions. Future studies should focus on prospective, standardized protocols with clear risk stratification, uniform outcome definitions, and direct comparison of adjunctive methods such as PRF, PBM, pentoxifylline with tocopherol, and drug holiday strategies. Until stronger evidence is available, prevention should remain individualized, interdisciplinary, and based on minimizing local infection, reducing surgical trauma, and optimizing systemic and metabolic conditions before and after tooth extraction. Patients with a history of AA therapy should be treated with caution. Antibiotic prophylaxis may be considered on an individualized basis, taking into account the patient’s risk profile and local circumstances, while oral hygiene and vitamin D status should be considered on an individualized basis according to the patient’s risk profile. Adjunctive methods such as APC, PBM, and the PENTO protocol may help reduce MRONJ risk but current evidence does not allow their systematic recommendation in all cases. A multidisciplinary approach, together with carefully planned pre- and postoperative care, is essential for safe tooth extraction in patients receiving AA. Optimal care lasts 14 days; therefore, it should be achieved by integrating these protocols into a comprehensive, patient-tailored preventive strategy rather than by applying them as isolated interventions.

Limitations: The literature was selected and evidence was classified by a single reviewer, which may have enabled subjective judgment and selection bias. Moreover, no formal risk-of-bias tool or GRADE assessment was applied or made because the manuscript was designed as a narrative rather than a systematic review.

## Figures and Tables

**Table 1:** Risk factors associated with MRONJ/BRONJ development [6,9,11,12,13].

Risk Factor	Higher Risk	Lower Risk
Type of medication	Cancer patients: DMB (≤6.9%) comparable with Zoledronate (≤18%) Osteoporotic patients: DMB (0.3%) [6]	Osteoporotic patients: BPs (0.02–0.05%), Romosozumab (0.03–0.05%) comparable with alendronate (0.05%)
Mean duration	>3 years	<3 years
Prior use of AA	yes	no
Sex	Female	Male
Age		<24—minimal risk
Dose	High dose	Low dose
Administration route	Intravenously	Orally
Concomitant diseases	Diabetes, Rheumatoid arthritis, Immunosuppression	None
Denture	Full prosthesis, Ill-fitting dentures	Partial prosthesis/none
Oral hygiene	Moderate/severe calculus, Periodontitis < 4 mm, gingivitis	Minimal deposits of dental plaque with no signs of gingivitis or periodontitis
Indications for treatment	Malignancies	Osteoporosis
Extraction site (MRONJ case distribution)	Mandible (75%)	Maxilla (25%)
Unhealthy lifestyle habits	Nicotine addiction, alcohol	None
CTX level	<100 μg/mL	>150 μg/mL
Medical treatment	Corticosteroids, chemotherapy, angiogenesis inhibitors	-

**Table 2:** Evidence profile of preventive measures according to study design and guideline support.

Preventive Measure	Highest Level of Available Evidence	Overall Evidence Appraisal	Key References
Risk stratification	Guidelines, cohort data	High (guideline consensus)	[6,9,11,12,13,14]
Dental optimization before AA therapy	Guidelines, observational	High (guideline consensus)	[6,14,17,18]
Local infection control/CHX	Guidelines, observational	Moderate	[6,14,17]
Atraumatic extraction + primary closure	Guidelines, large case series	Moderate–high	[6,14,20,21]
Antibiotic prophylaxis	1 scoping review, 1 retrospective cohort, national guidelines	Moderate; mixed	[22,23,24,25,26]
APC (PRF/L-RF/PRP)	Meta-analysis, 1 systematic review non-significant, multiple observational	Low–moderate	[19,27,28,29,30,31,32,34,35]
Photobiomodulation	Case series, animal studies, systematic review with heterogeneity	Low	[39,40,42,43,44,45,46,47,48]
PENTO protocol	Small case series, literature reviews of treatment	Very low	[51,52,53,54,55]
Vitamin D supplementation	Hypothesis papers, indirect evidence	Very low	[56,57,59,60,61]
Drug holiday	1 RCT feasibility trial, animal, cohort	Low; controversial	[3,62,63,65,66,67]
Hyperbaric oxygen therapy	Extrapolation from ORN, animal studies	Very low	[68,69,70,71,72,73,74]

**Table 3:** Prevention methods depending on risk.

Prevention Method/ Specific Group of Patients	MRONJ Cancer Patients	MRONJ Non-Cancer Patients Higher Risk/Lower Risk	BRONJ Cancer Patients	BRONJ Non-Cancer Patients Higher Risk/Lower Risk
Antibiotic	1 g amoxicillin with clavulanic acid for 14 days	1 g amoxicillin with clavulanic acid for 14 days/1 day before procedure and 3 subsequent days after the procedure	1 g amoxicillin with clavulanic acid for 14 days	1 g amoxicillin with clavulanic acid for 14 days/1 day before the procedure and 3 subsequent days after the procedure
Oral hygiene	Especially Optimize dental health, 6-monthly dental check-ups, 0.2% chlorhexidine gluconate (ChG) 7 days following extraction	Optimize dental health, regular 6-monthly dental check-ups, 0.2% ChG for 7 days following the extraction	Especially Optimize dental health, 6-monthly dental check-ups, 0.2% ChG for 7 days following the extraction	Optimize dental health, 6-monthly dental check-ups, 0.2% ChG for 7 days following the extraction
APCs	L-PRF or PRP	L-PRF or PRP/facultative	L-PRF or PRP	L-PRF or PRP/facultative
LLLT	Nd:YAG or diode laser	Nd:YAG or diode laser/facultative	Nd:YAG or diode laser	Nd:YAG or diode laser/facultative
Drug suspension	-	DMB: 3–4 months before the procedure and resuming 2 months after it	4 months before procedure and resuming 2 months after it	4 months before procedure and resuming 2 months after it (osteoporosis: if fracture risk is low)
PENTO	Adjunctive 400 mg pentoxifylline and 400 mg tocopherol 2 times per day	Adjunctive 400 mg pentoxifylline and 400 mg tocopherol 2 times per day	Adjunctive 400 mg pentoxifylline and 400 mg tocopherol 2 times per day	Adjunctive 400 mg pentoxifylline and 400 mg tocopherol 2 times per day
Vitamin D supplementation	0.75 μg Eldalcitol/day	0.75 μg Eldalcitol/day	0.75 μg Eldalcitol/day	0.75 μg Eldalcitol/day

## Data Availability

No new data were created or analyzed in this study. Data sharing is not applicable to this review article.

## Abbreviations

The following abbreviations are used in this manuscript:

BPs	Bisphosphonates
DMB	Denosumab
ZOL	Zoledronic Acid
AA	Antiresorptive Agent
PRF	Platelet-Rich Fibrin
CGF	Concentrated Growth Factors
LLLT	Low-Level Laser Therapy
ONJ	Osteonecrosis of Jaw
BRONJ	Bisphosphonate-Related Osteonecrosis of Jaw
MRONJ	Medication-Related Osteonecrosis of Jaw
AAOMS	American Association of Oral and Maxillofacial Surgeons
TKIs	Tyrosine Kinase Inhibitors
CTX	Beta-C-Terminal Telopeptide
APCs	Autologous Platelet Concentrates
PRP	Platelet-Rich Plasma
L-PRF	Leukocyte- and Platelet-Rich Fibrin
GelMA	Methacrylated Gelatin
HepMA	Methacrylated Heparin
PBM	Photobiomodulation
OPG	Osteoprotegerin
PENTO protocol	Pentoxifylline and Tocopherol Protocol
HBO	Hyperbaric Oxygen Therapy
ASCO	An American Society of Clinical Oncology Journal
ORN	Osteoradionecrosis
ChG	Chlorhexidine Gluconate
